# MICROBIOME: The trials and errors of developing an experimental model to study the impact of maternal gut microbiome disruption on perinatal asphyxia

**DOI:** 10.1530/RAF-24-0050

**Published:** 2024-11-06

**Authors:** Mara Ioana Ionescu, Ana Maria Catrina, Ioana Alexandra Dogaru, Didina Catalina Barbalata, Cristian Ciotei, Cerasela Haidoiu, Vladimir Suhaianu, Gratiela Gradisteanu Pircalabioru, Siobhain M. O’Mahony, Ana-Maria Zagrean

**Affiliations:** 1 Division of Physiology II – Neuroscience, Department of Functional Sciences, Faculty of Medicine, Carol Davila University of Medicine and Pharmacy, Bucharest, Romania; 2 Department of Pediatrics, Marie Curie Emergency Children's Hospital, Bucharest, Romania; 3 Cantacuzino National Military Medical Institute for Research and Development, Bucharest, Romania; 4 Research Institute of the University of Bucharest, Section Earth, Environmental and Life Sciences, Section-ICUB, Bucharest; 5 eBio-Hub Research-Center, National University of Science and Technology “Politehnica” Bucharest, Campus Building, Bucharest, Romania; 6 Department of Anatomy and Neuroscience, University College Cork, Cork, Ireland; 7 APC Microbiome Ireland, University College Cork, Cork, Ireland

**Keywords:** gestational antibiotic administration, gut-brain axis, maternal gut microbiome, neurodevelopment, perinatal asphyxia

## Abstract

**Abstract:**

Maternal gut microbiome impairment has garnered attention for its potential role in influencing neurodevelopmental outcomes in offspring, especially in situations that increase brain vulnerability such as perinatal asphyxia (PA). Maternal microbiome and fetal brain interplay emerge as a critical link between maternal health and offspring neurodevelopment. This study aims to generate a model to assess the impact of maternal dysbiosis triggered by gestational antibiotic administration and PA on offspring neurodevelopment. Wistar rats were subjected to antibiotics in drinking water from the 11th gestational day until birth. On the 6th postnatal day, pups were subjected to PA/normoxia, resulting in four experimental groups: control-normoxia, antibiotics-normoxia, control-asphyxia, and antibiotics-asphyxia. Early-life behavioral tests were conducted between postnatal days 7 and 9. The initial antimicrobial cocktail (ampicillin, vancomycin, neomycin, clindamycin, amphotericin-B) led to an increased number of miscarriages, poor weight gain during pregnancy, reduced offspring weight, and changes in the maternal gut microbiome compared to control. Offspring presented impaired neurodevelopmental reflexes in both PA and antibiotic groups and increased hippocampal neuroinflammation. Due to these detrimental effects, a more pregnancy-safe antibiotic cocktail was used for a second experiment (ampicillin, vancomycin, neomycin, meropenem). This resulted in no miscarriages or pregnancy-weight loss but was still linked to gut microbiome disruption. PA impaired neurodevelopmental reflexes and increased neuroinflammation, effects amplified by antibiotic administration. These preliminary findings reveal the cumulative potential of maternal dysbiosis and PA on neurodevelopment impairment, emphasizing caution in gestational antimicrobial use. Further investigations should include offspring long-term follow-up and maternal behavior and integrate probiotics to counteract antibiotic effects.

**Graphical abstract:**

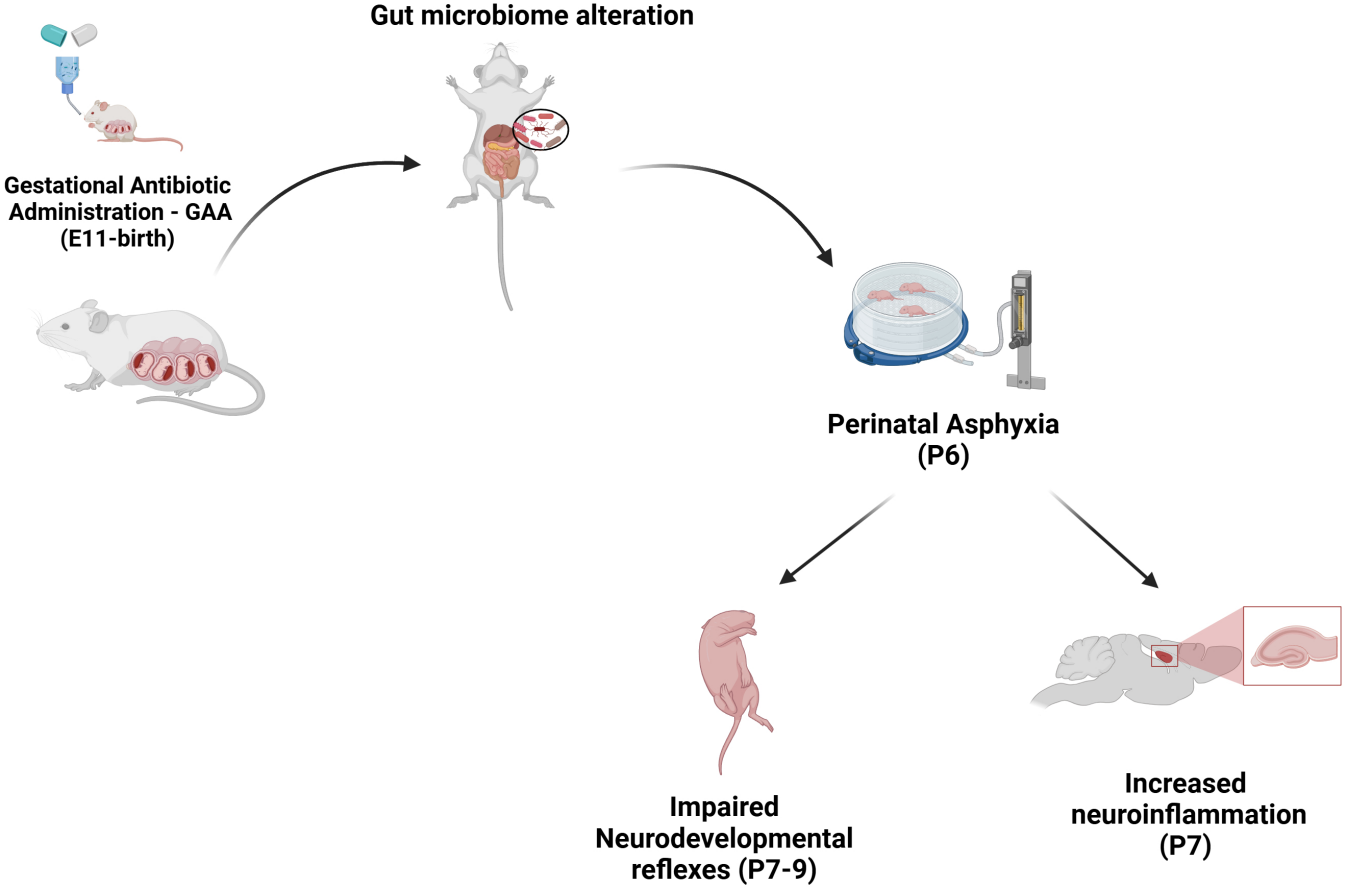

**Lay summary:**

This study investigates the impact of maternal gut microbiome disruptions caused by gestational antibiotic treatment and low oxygen exposure shortly after birth on the development of the rats’ babies. We found that both antibiotic exposure and reduced oxygen levels led to changes in early behavior and increased inflammation of the nervous tissue in the baby rats. Although using a different, potentially safer antibiotic combination reduced pregnancy complications, it still changed the bacteria in the mother’s gut and worsened early behavior. These findings show that antibiotics during pregnancy can affect the developing brain of baby rats and careful consideration should be used before prescribing them. Future research will explore longer-term effects and potential medicines.

## Introduction

The gut microbiota represents a highly intricate ecosystem crucial for maintaining host homeostasis, influenced by various exogenous and intrinsic factors that are not yet fully understood. Dysbiosis, or gut microbial imbalance, has been implicated in several pathological conditions, highlighting the importance of maintaining eubiosis, or a balanced gut microbiota composition, through tailored dietary interventions and antibiotic treatments (Dudek-Wicher *et al.* 2018).

Parturition is accompanied by a transient period of asphyxia characterized by hypoxia and hypercapnia in the neonate, constituting a normal physiological response to birth stress. However, prolonged birth asphyxia, resulting from extended labor or umbilical occlusion, can lead to severe short- and long-term consequences, particularly affecting the vulnerable and hypoxia-sensitive immature brain (Gillam-Krakauer *et al.* 2024). Perinatal asphyxia (PA) is a significant contributor to neonatal and childhood mortality and morbidity worldwide, particularly associated with conditions such as hypoxic-iIschemic encephalopathy (HIE), predisposing individuals to neurological and psychiatric disorders (Lawn *et al.* 2011).

Maternal factors, such as diets, play a role in modulating the severity and impact of PA. A high-fat diet has been found to increase neonatal susceptibility (Isac *et al.* 2018), while supplementing with certain nutrients like trans-resveratrol and citicoline has neuroprotective effects (Isac *et al.* 2017, Isac *et al.* 2020). Considering the pivotal role of diet in shaping the diversity and composition of the gut microbiota, we hypothesize that the maternal gut microbiome plays a significant role in determining the vulnerability of the offspring's immature brain to PA.

The gut microbiota and the brain undergo rapid and parallel maturation during early development, with significant interactions between them. The notion that maternal gut bacteria can influence both the development of the infant’s brain and their gut microbiota during pregnancy has been proposed (Codagnone *et al.* 2019). Alterations in the maternal gut microbiome due to factors such as infection (Kim *et al.* 2017), dietary changes (Ionescu *et al.* 2024), stress (Golubeva *et al.* 2015), and antibiotic therapy (Vuong *et al.* 2020), have been associated with abnormalities in offspring brain function and behavior yet the exact mechanism is not clear. Furthermore, the concept of an *in utero* or fetal microbiome has been significantly challenged hence direct microbial effects on the offspring during pregnancy are unlikely yet there is scope for circulating metabolites (Kennedy *et al.* 2023).

Epidemiological studies have shown that early-life exposure to antibiotics can increase the risk of developing immune and metabolic diseases (Lamont *et al.* 2020). In rodents, antibiotic administration to dams results in behavior deficits in the offspring, but with conflicting outcomes regarding activity, social interactions, and anxiety (O’Connor *et al.* 2021). Also, the expression of genes associated with axonogenesis is impaired in mouse embryos from antibiotic-treated and germ-free dams leading to altered neurodevelopment and behavior (Vuong *et al.* 2020).

Germ-free animal models have been instrumental in elucidating the gut microbiome's influence on the brain, but they lack clinical relevance. Therefore, using orally administered antibiotics to induce dysbiosis in holoxenic animals provides a more translational approach to studying the effects of the intestinal microbiome on neurodevelopment (Kennedy *et al.* 2018). Although a couple of studies explored the influence of maternal antibiotic-mediated dysbiosis on the offspring’s behavior and neurodevelopment, no studies have created a reproducible model to study maternal antibiotic-mediated dysbiosis in PA.

The novelty of our model relies on the association of two common conditions: (i) maternal gut microbiome disruption during pregnancy, which is known to impact the offspring’s neurological function and structure; (ii) the transient period of asphyxia that accompanies parturition. This is the first endeavor to create an experimental *in vivo* model that investigates the influence of maternal gut microbiota antibiotic disruption on the offspring’s brain tolerance to PA.

## Materials and methods

### Animals

The experiments were conducted on Wistar rats provided with access to a standard diet and water *ad libitum*. All animal procedures were carried out with the approval of the local ethics committee for animal research following the European Communities Council Directive 86/609/EEC on the protection of animals used for scientific purposes and approved by the Animal Experimentation Ethics Committee of Cantacuzino National Military Medical Institute for Research and Development (37/23.08.2023) and of Carol Davila University of Medicine and Pharmacy (21994/02.08.2022). The rats (aged 90–100 days) were brought in and mated in our Biological Services Unit (Cernica Biological Service Unit of Cantacuzino National Military Medical Institute for Research and Development and the Biological Service Unit of Carol Davila University of Medicine and Pharmacy). The number of used rats varied based on the experiment.

For experiment 1, 39 female rats (15 control group, 24 antibiotic group) were mated with 6 male rats, and treated with a first antibiotic cocktail starting with the gestational day 10–11, as appointed by an experienced veterinary doctor, until birth. Of the 39 female rats, 26 gave birth to live pups, 14 from the control group, and 12 from the antibiotic group. Subsets of the litter were exposed to asphyxia or normoxia, and the whole litter of pups was either sacrificed for further sample analysis or subjected to neurodevelopmental reflexes.

**Figure 1:**
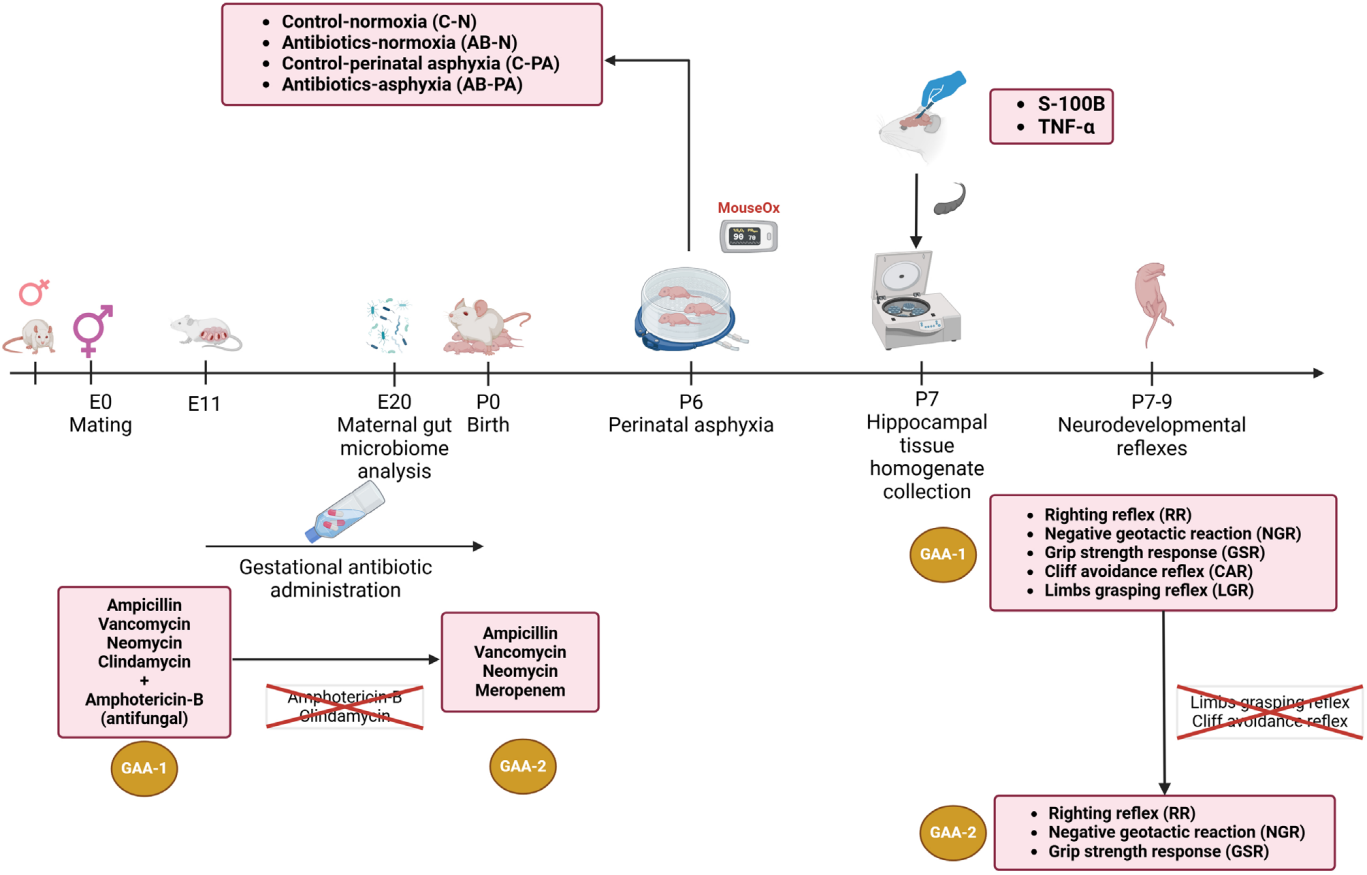
Flowchart of the overall experimental design. E, embryonic day; GAA, gestational antibiotic administration; P, postnatal day.

For experiment 2, 8 female Wistar rats (4 control group, 4 antibiotic group) were mated with 4 male Wistar rats. The gestational day 0 was considered based on the vaginal plug visualization, and treatment with a second antibiotic cocktail started at gestational day 10–11 until birth. After birth, part of the litter was exposed to asphyxia or normoxia, and part of the litter was sacrificed or subjected to neurodevelopmental reflexes. The overall experimental design is illustrated in Fig. 1.

## Gestational antimicrobial administration

### Experiment 1: gestational antimicrobial administration-1 (GAA-1)

To efficiently deplete the gut microbiota during pregnancy a cocktail of antibiotics (ampicillin 1 mg/mL, vancomycin 1 mg/mL, neomycin 5 mg/mL, clindamycin 1 mg/mL) was orally administered in the drinking water, as modified after previous studies in the literature (Lamousé-Smith *et al.* 2011, Bookstaver *et al.* 2015, Desbonnet *et al.* 2015, Fröhlich *et al.* 2016, Tochitani *et al.* 2016, Nyangahu *et al.* 2018, Vuong *et al.* 2020, O’Connor *et al.* 2021, Bongers *et al.* 2022, Faas *et al.* 2023). Due to the bitter taste of metronidazole, this antibiotic was switched to clindamycin (Lamousé-Smith *et al.* 2011, Reikvam *et al.* 2011, Bongers *et al.* 2022). Among the antibiotics used, neomycin and vancomycin have low oral bioavailability, potentially reducing systemic toxicity (Fröhlich *et al.* 2016, de Bruijn *et al.* 2020). Additionally, ampicillin and vancomycin have been deemed safe for use during gestation (Bookstaver *et al.* 2015). To prevent fungal growth, amphotericin-B 0.1 mg/mL was also added to the antibiotic cocktail (Desbonnet *et al.* 2015), completing the first gestational antimicrobial combination. Due to its insolubility in water (Adediran *et al.* 2009), amphotericin-B was diluted using a small amount of dimethyl sulfoxide (DMSO) solvent, resulting in a final DMSO concentration of 1%, deemed to be safe (Hoyberghs *et al.* 2021).

### Experiment 2: gestational antibiotic administration-2 (GAA-2)

To address the adverse effects observed in dams receiving GAA-1, such as elevated incidence of miscarriages and low offspring weight, we undertook refinement of the antibiotic cocktail. Given the conflicting evidence in the literature regarding the teratogenic potential of Amphotericin-B (Khosravi *et al.* 2022) and DMSO (Cheng *et al.* 2022), and the high oral bioavailability of clindamycin (Batzias *et al.* 2005), these were omitted from GAA-2 formulation. In the previous study that used this antifungal supplementation, the antibiotic cocktail was administered for a long period (60 days) (Desbonnet *et al.* 2015). Therefore, due to the short antibiotic administration in our study, we stopped administering the amphotericin-B. Furthermore, aiming for antibiotics with a greater impact on the gut microbiome, we replaced clindamycin, known for its high oral bioavailability, with meropenem, which exhibits lower oral bioavailability (Raza *et al.* 2021). Consequently, the revised antibiotic cocktail was composed of ampicillin (1 mg/mL), vancomycin (0.5 mg/mL), neomycin (5 mg/mL), and meropenem (1 mg/mL) (Fröhlich *et al.* 2016, O’Connor *et al.* 2021, Deng *et al.* 2023, Faas *et al.* 2023).

The treatment for both experiments was given starting approximately with gestational day 11, which corresponds to the neural tube formation in rats (Semple *et al.* 2013) until birth. The water was changed every 2 days for experiment 1 and daily for experiment 2. To advance our investigation into probiotic supplementation, we opted to administer the GAA-2 regimen during nighttime (16:00 h – 08:00 h), reserving the daytime period for probiotic intervention, a model developed after a previous study (Leclercq *et al.* 2017).

Maternal weights were measured regularly and glucose levels were determined close to the birth date. Liquid intake was estimated by measuring bottle weights before replenishment. Water intake in each cage was closely monitored to ascertain no notable variances among the treatment groups.

### Perinatal asphyxia exposure

After birth, pups were divided into two groups: control (C) and antibiotic (AB) with pups coming from at least 2 litters/group. On postnatal day (P) 6, whole litters of pups (C and AB) were assigned either to the normoxia group (N) or perinatal asphyxia group (PA) by selecting them randomly from different females. Using a birth asphyxia paradigm developed by Helmy (Helmy *et al.* 2012) and further modified by our group, pups were exposed for 90 min to either normoxia or asphyxia (9% O_2_, 20% CO_2_ in N_2_) (Panaitescu *et al.* 2018). An open non-rebreathing system delivered the asphyxia gas combination at a constant flow rate of 2 L/min. The asphyxia exposure was assessed using a MouseOx monitor to dynamically collect the offspring’s vital signs and data regarding heart rate, arterial blood saturation, breath rate, and temperature during PA exposure. The temperature was maintained at 37°C during exposure using a heating pad (FHC Inc., Bowdoin, USA). All the pups were returned to their mothers immediately after exposure. The resulting four experimental groups were: control-normoxia (C-N), antibiotics-normoxia (AB-N), control-perinatal asphyxia (C-PA), and antibiotics-perinatal asphyxia (AB-PA).

### Neurodevelopmental reflexes

To explore the early effects of antibiotics and asphyxia on offspring behavior, a subset of pups (males and females) underwent neurodevelopmental reflex testing from P7 to P9, while the others were sacrificed for further sample analysis. Given the limitations of exploring more complex behavior assessments at this developmental stage, the neurodevelopmental reflexes were investigated (Nguyen *et al.* 2017). No sex differences between the pups were observed at this stage.

Experiment 1 (GAA-1)**:** Starting 24 h post-asphyxia, between P7 and P9, pups from each litter were subjected to early-life behavioral testing, including the righting reflex (RR), the negative geotactic reaction (NGR), the grip strength response (GSR), the cliff avoidance reflex (CAR), and the limbs grasping reflex (LGR).

#### The righting reflex (RR)

The neonate rat was placed on its back on a flat surface and the time required for the pup to right itself through 180° was measured. If necessary, each trial was allocated a maximum duration of 1 min, and the test was repeated three times, with the average time recorded (Nguyen *et al.* 2017).

#### The negative geotactic reaction (NGR)

The neonate pup was placed head down on a surface inclined at 45°. Each pup was observed for 180 s to turn and move toward the upper end of the surface. Motor and vestibular input is required for the mouse to recognize its orientation on a slope and turn around. The process was repeated for a total of three trials. Pups that didn’t turn or fell down the inclined plane were given a zero score (Ruhela *et al.* 2019).

#### The grip strength response (GSR)

For this test, a screen wire was used. Pups were placed on this screen at a horizontal level and allowed to accommodate for 5 s. Afterward, the screen was slowly inverted to 180°. The approximate angle of the screen was recorded when the pup fell. The procedure was repeated three times, and subsequently, the results were averaged (Feather-Schussler & Ferguson 2016).

#### The cliff avoidance reflex (CAR)

This test assesses the protective response of the pup when its snout and forepaw are placed on the edge of a flat surface. The pup was placed on a box with a flat elevated ledge, with only the digits of their forepaws and their snout positioned over the side. The duration required for the pup to turn away from the cliff while retracting its paws and snout away from the edge was used to calculate the score. The test was canceled if there was no response after 30 s. The procedure was repeated 3 times. There was no score if the pup didn’t move away from the cliff within 30 s (Nguyen *et al.* 2017).

#### The limbs grasping reflex (LGR)

This test was conducted by placing a blunt rod against the palm/planta of each forepaw, exerting light pressure. Grasping appeared as flexion of all digits around the rod. This test could be repeated until the appearance of the reflex (Nguyen *et al.* 2017). As it is a reflex, it is impossible to learn.

Experiment 2 (GAA-2):To refine and capture the most relevant data during the neonatal behavior assessments, we performed three selected neurodevelopmental reflex tests over 3 consecutive days, commencing 24 h post-asphyxia exposure. Given its subjective nature, we omitted the limbs grasping reflex test from our protocol. Instead, we retained the negative geotactic reaction and grip strength response tests, which yielded statistically significant results, along with the widely utilized righting reflex test.

### Hippocampal tissue homogenate collection

At 24 h following asphyxia/normoxia exposure, subsets of offspring from all experimental cohorts were culled in accordance with animal ethical guidelines, and their hippocampi were dissected in ice-cold conditions, isolated from meningeal structures, and subsequently rinsed in ice-cold PBS (0.02 mol/L, pH = 7.0–7.2). The hippocampal tissue was then finely minced, homogenized using a glass homogenizer, subjected to sonication for 10 min, and centrifuged for 5 min at 5000 ***g*** and 4°C. Following centrifugation, the supernatant was carefully extracted and stored at −80°C until all samples were collected and prepared for analysis.

### ELISA evaluation of neuroinflammatory and neural injury biomarkers

Using the ELISA technique performed on Multimodal Microplate Reader EnSight (Perkin Elmer) and following the manufacturer’s recommendations, the levels of TNF-α (Abcam ab100785), S-100B (Abcam ab234573), and total protein content (Lowry method) were determined in the hippocampal tissue homogenate. TNF-α and S-100B levels were quantified and expressed as ratios relative to the total protein content (pg/μg protein).

### Maternal fecal collection and blood glucose levels during pregnancy

On gestational day 20 ± 1 days, fecal samples from the pregnant dams were collected and stored at −80°C until further analysis. Additionally, blood from the dams’ tail was collected during the antibiotic exposure and the glucose levels were checked.

### Gut microbiome analysis

DNA extraction was conducted with the QIAamp DNA Stool Mini Kit (Qiagen) as per the manufacturer’s protocol. The concentration of DNA was determined using the Qubit Broad Range kit and the Qubit 4 fluorometer (Thermo Scientific). For quantitative Real-Time PCR analysis, microbial DNA samples were diluted to a concentration of 3 ng/μL. Bacterial group-specific 16S rRNA primers were employed for microbiota analysis via qRT-PCR, with primer sequences provided in Table 1. Each amplification reaction comprised 9 ng of DNA, 2.5 nM primers, and SYBR Green Master Mix (Applied Biosystems). Samples were analyzed using a ViiA7© Fast Real-Time instrument (Applied Biosystems).

**Table 1:** Primer sequences 5′-3′ orientation.

*Bacteroides* spp.	cctacgatggataggggtt
	cacgctacttggctggttcag
*Butyricicoccus* spp.	acctgaagaataagctcc
	gataacgcttgctccctacgt
*BPP*	ggtgtcggcttaagtgccat
	cggacgtaagggccgtgc
*Lactobacillus* spp.	acgagtagggaaatcttcca
	caccgctacacatggag
*Fusobacterium* spp.	acctaagggagaaacagaacca
	cctgcctttaattcatctccat
*Faecalibacterium* spp.	cccttcagtgccgcagt
	gtcgcaggatgtcaagac
*Enterobacteriaceae*	gtgccagcmgccgcggtaa
	gcctcaagggcacaacctcc ag
*Sutterella* spp.	cgcgaaaaaccttacctagcc
	gacgtgtgaggccctagcc
*Bifidobacterium* spp.	gggtggtaatgccggatg
	ccaccgttacaccgggaa

### Statistical analysis

The data were analyzed using the statistical software package GraphPad Prism 9.0, and the results were presented as mean ± standard error of the mean (s.e.m.). Data were assessed for normality using the Shapiro–Wilk test. Normally distributed data were analyzed using a *t*-test or two-way ANOVA depending on the data, and non-normally distributed data were analyzed using the Mann–Whitney test. The threshold for statistical significance was set at a *P*-value of 0.05. The ‘*n*’ value represents the number of animals per experimental group.

## Results

### GAA-1 induces a detrimental reduced maternal weight gain throughout pregnancy with increased miscarriages, whereas GAA-2 does not exhibit a similar adverse impact

**Figure 2:**
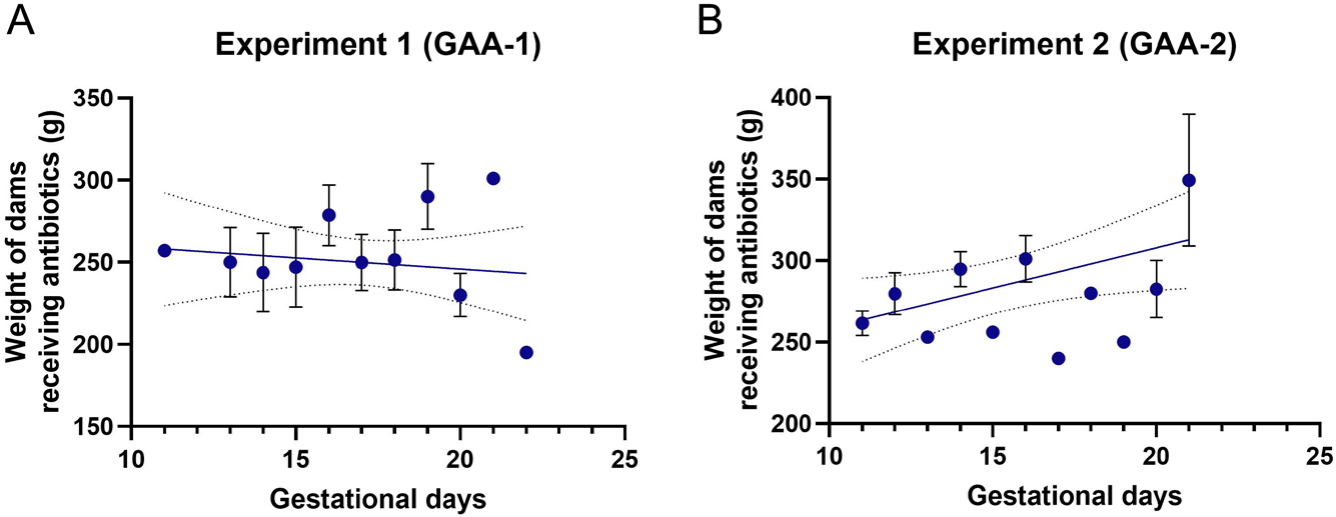
The weight variation in dams exposed to gestational antibiotic administration during pregnancy: (A) In experiment 1, GAA-1 led to a slight decrease of weight during the pregnancy period (p_slope_ = −1.353, R^2^ = 0.010, *P* = 0.436); (B) In experiment 2, GAA-2 led to a normal increase in dams’ weight during pregnancy (p_slope_ = 4.904, R^2^ = 0.195, *P* = 0.309). The control group animals exhibited consistent weight gain throughout pregnancy. GAA, gestational antibiotic administration.

In experiment 1, GAA-1 resulted in a notable deviation from the expected weight trajectory during pregnancy, with a discernible decrease observed (p_slope_ = −1.353, R^2^ = 0.010, *P* = 0.436) (Fig. 2A). Conversely, in experiment 2, GAA-2 administration did not elicit a similar effect, as dams exhibited a pattern of weight gain consistent with normal physiological expectations throughout the gestational period (p_slope_ = 4.904, R^2^ = 0.195, *P* = 0.309) (Fig. 2B). The animals in the control group exhibited consistent weight gain throughout pregnancy.

**Figure 3:**
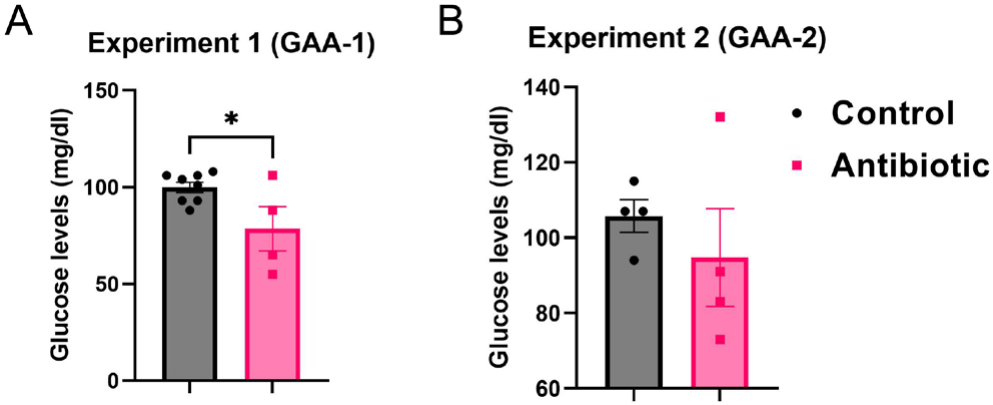
Glucose levels in dams collected during pregnancy, close to the birth date, after antibiotic initiation: (A) In experiment 1, GAA-1 (*n* = 4) led to a decrease in glucose levels when compared to the control group (*n* = 8, *P = 0.015); (B) In experiment 2, GAA-2 (*n* = 4) didn’t significantly impact the glucose levels in dams compared to the ones from the control group (*n* = 4, *P* = 0.106). Results are expressed as means ± SEM. *P < 0.05. GAA, gestational antibiotic administration.

The glucose levels in dams during pregnancy following antibiotic administration were modified differently in the two experiments. In experiment 1, after GAA-1 initiation, a reduction in glucose levels was observed compared to the control group (78.500 ± 11.480 mg/dL, *n* = 4 vs 99.880 ± 2.655 mg/dL, *n* = 8, *P = 0.015) (Fig. 3A). In experiment 2, GAA-2 did not significantly alter the glucose levels in dams compared to those in the control group (94.750 ± 12.950 mg/dL, *n* = 4 vs 105.8 ± 4.347 mg/dL, *n* = 4, *P* = 0.106) (Fig. 3B).

**Figure 4:**
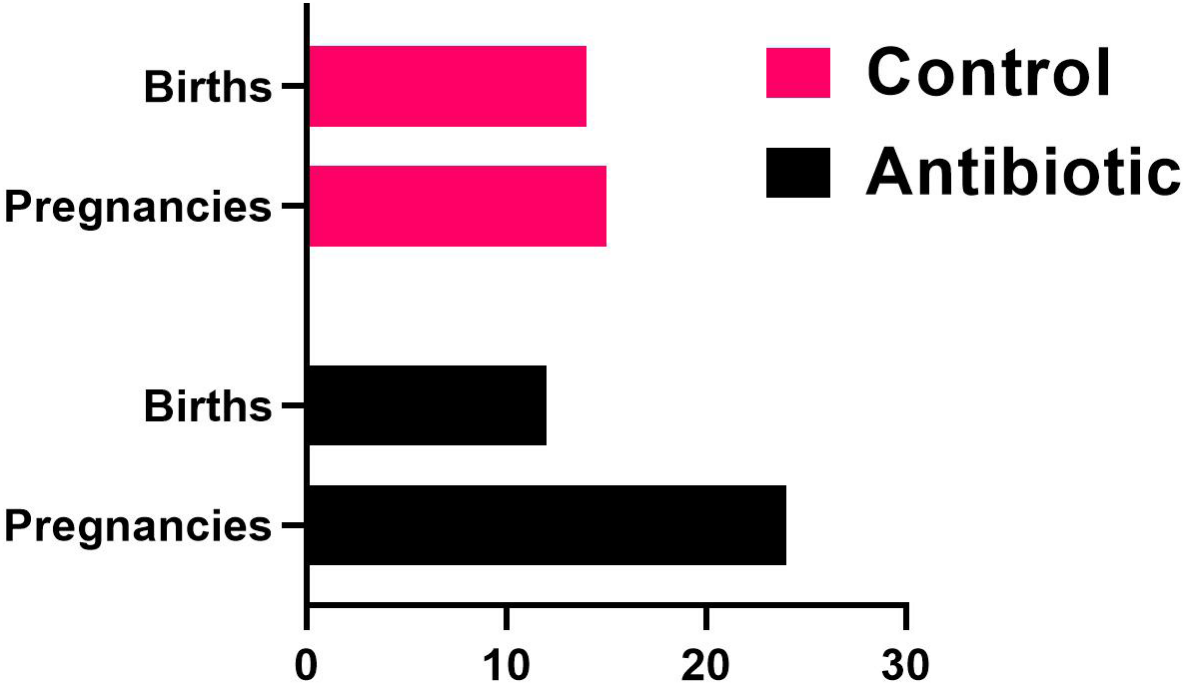
Offspring survival rates were compared between the two groups, control, and antibiotic, with the x-axis representing the number of animals. Administration of prenatal antimicrobials (GAA-1) resulted in a higher incidence of stillbirths, reaching 50%.

Prenatal administration of antimicrobials (GAA-1) resulted in a heightened incidence of miscarriages, observed at a rate of 50% (Fig. 4). Conversely, GAA-2 did not elicit a similar effect, as dams exhibited appropriate weight gain during pregnancy, and litters were characterized by a high number of pups (15 in the control group, 9 in the antibiotic group).

### GAA-1 leads to reduced weight in offspring relative to control

**Figure 5:**
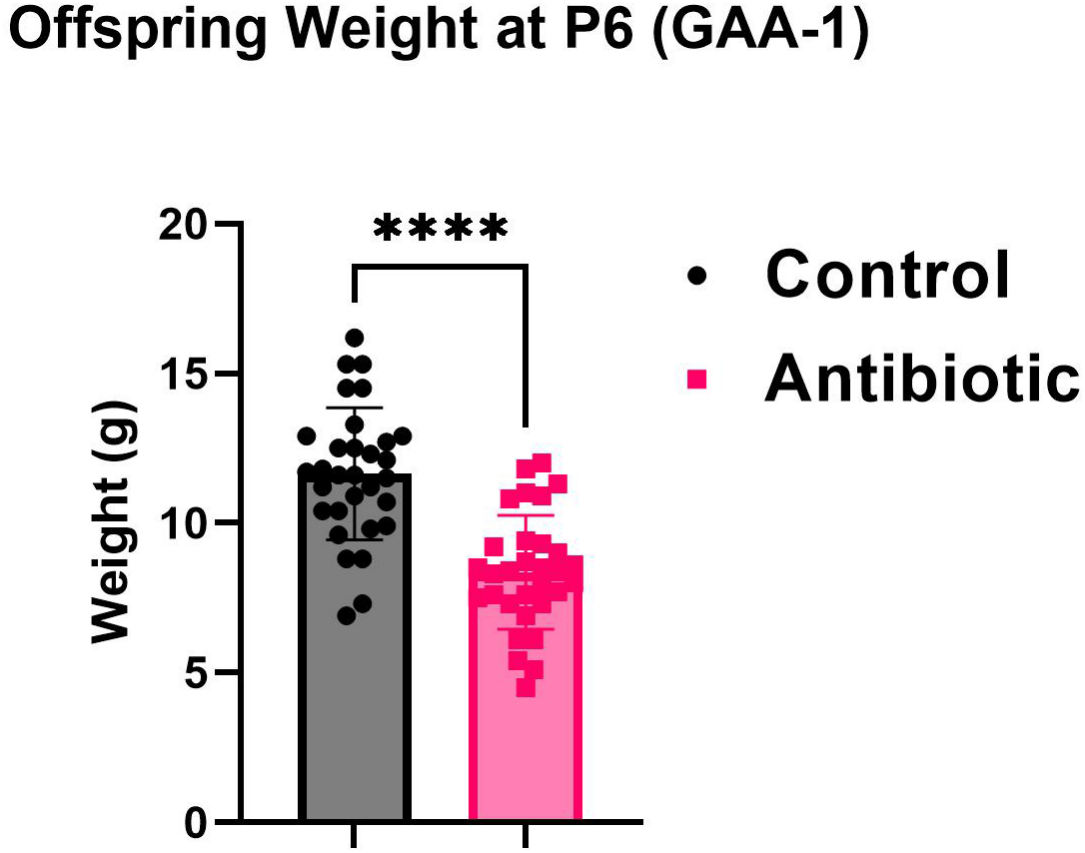
The offspring’s weight at postnatal day 6 after the GAA-1 compared to the control. GAA-1 significantly decreased the offspring weight (*n* = 31) compared to the control group (*n* = 31, ****P < 0.0001). No difference was observed for GAA-2 (data not shown). Results are expressed as means ± SEM. ****P < 0.0001. GAA, gestational antibiotic administration.

The assessment of offspring weight at P6 following GAA-1 was conducted to ascertain any discernible differences compared to the control cohort. Notably, GAA-1 exposure led to a statistically significant reduction in offspring weight (8.358 ± 0.340 g, *n* = 31) relative to the control group (11.65 ± 0.396 g, *n* = 31, ****P < 0.0001) (Fig. 5), indicating a tangible impact of the administered antibiotic regimen on postnatal growth. In experiment 2, GAA-2 did not induce any changes in the offspring weight (data not shown).

### Antibiotic administration during pregnancy alters the gut microbiome in dams

#### Experiment 1 (GAA-1)

**Figure 6:**
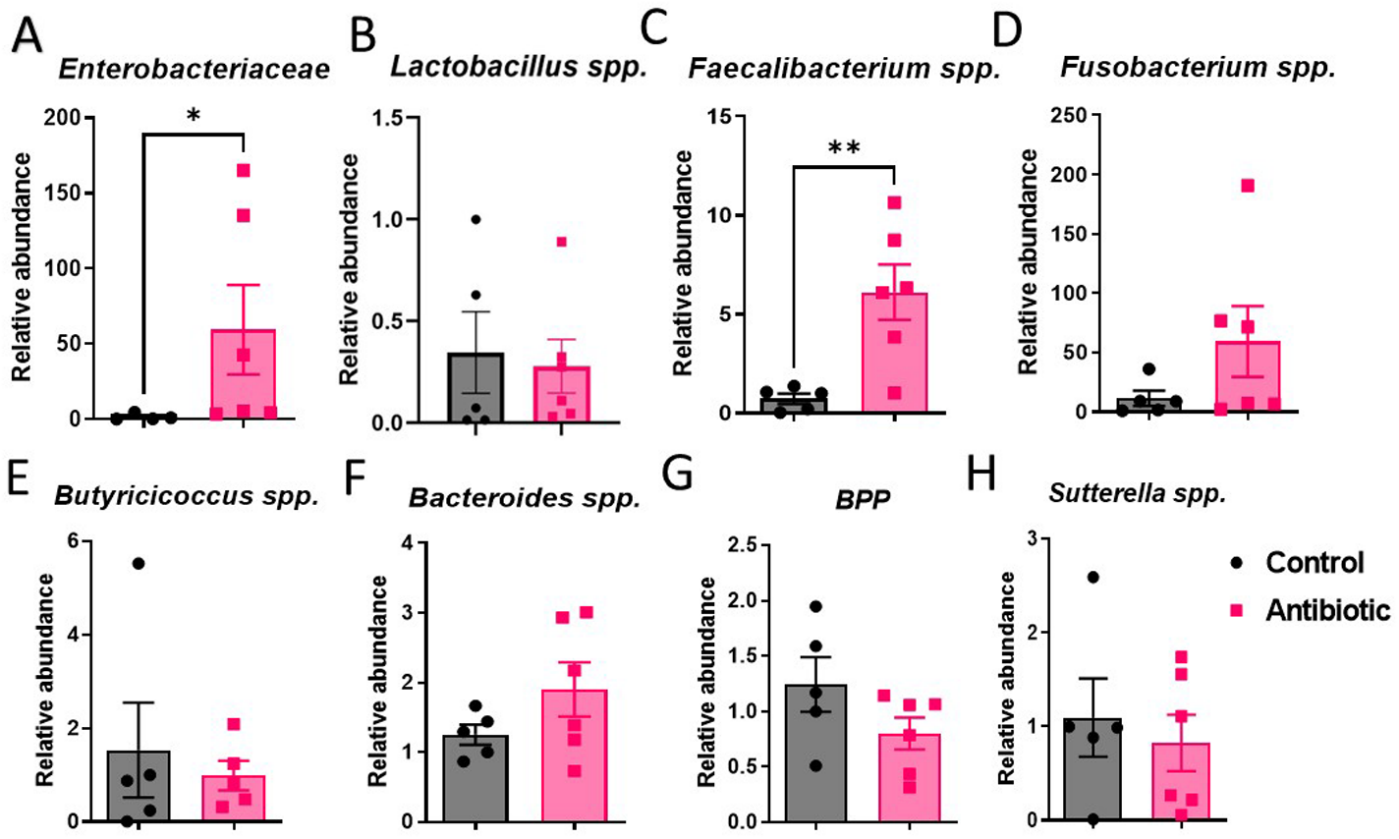
The relative abundance of (A) *Enterobacteriaceae*, (B) *Lactobacillus* spp., (C) *Faecalibacterium* spp., (D) *Fusobacterium* spp., (E) *Butyricicoccus* spp., (F) *Bacteroides* spp., (G) BPP (Bacteroides–Prevotella–Porphyromonas), (H) *Sutterella* spp. in AB group compared to C group in experiment 1. Results are expressed as means ± SEM. *P < 0.05, **P = 0.01–0.05.

In experiment 1, there were no considerable changes observed in the gut microbiome of the antibiotic-exposed rats compared to the control group, possibly attributed to the limited sample size (Fig. 6). However, the relative abundance of *Enterobacteriaceae* was notably elevated in the antibiotic-exposed group in comparison to the control group (*P = 0.038) (Fig. 6A). Intriguingly, the relative abundance of *Faecalibacterium* spp*.* was significantly higher in the antibiotic-exposed group compared to the control group (**P = 0.007) (Fig. 6C). Notably, the antibiotics’ administration did not appear to exert a substantial effect on the abundance of *Lactobacillus* spp*.* (Fig. 6B), likely due to the relatively modest available sample size. Furthermore, trends in relative abundances were discerned: antibiotic administration was associated with increased relative abundances of *Fusobacterium* spp. (Fig. 6D) and *Bacteroides* spp*.* (Fig. 6F), along with decreased levels of *Butyricicoccus* spp*.* (Fig. 6E)*, BPP* (Fig. 6G), and *Sutterella* spp*.* (Fig. 6H) compared to control.

#### Experiment 2 (GAA-2)

**Figure 7:**
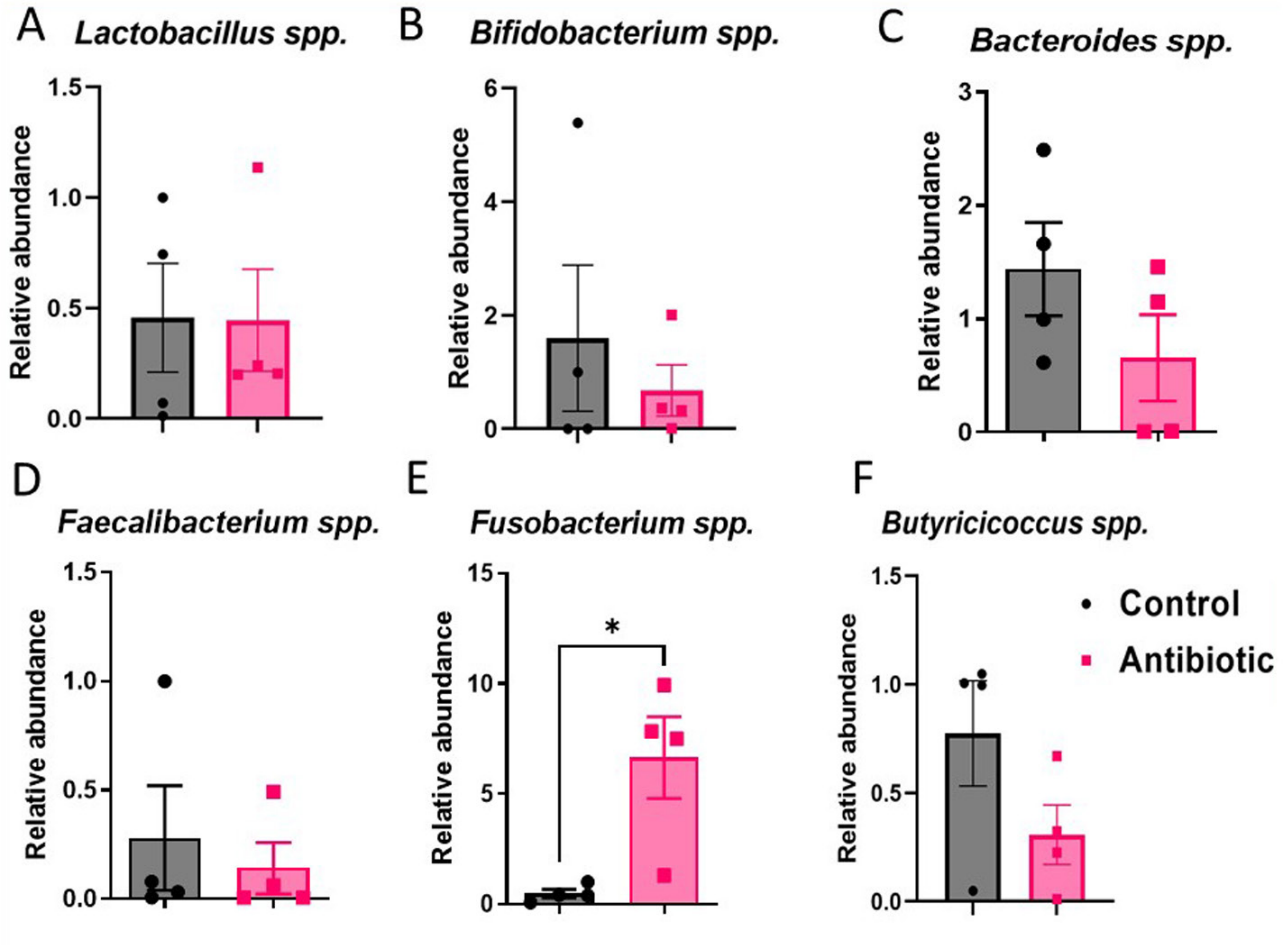
The relative abundance of (A) *Lactobacillus* spp., (B) *Bifidobacterium* spp., (C) *Bacteroides* spp., (D) *Faecalibacterium* spp., (E) *Fusobacterium* spp., (F) *Butyricicoccus* spp. in AB group compared to the C group in experiment 2 - preliminary results due to the low number of samples. Results are expressed as means ± SEM. *P < 0.05.

In experiment 2, antibiotic exposure did not elicit substantial alterations in the gut microbiome, likely due to the limited sample size (Fig. 7). However, among the antibiotic-treated dams, the relative abundance of *Fusobacterium* was significantly higher compared to the control group (*P = 0.016) (Fig. 7E). Notably, antibiotic exposure did not significantly affect the levels of *Lactobacillus* (Fig. 7A), also possibly due to the constrained sample size utilized in the study. Furthermore, trends in the relative abundances of the antibiotic-treated group indicated reductions in the levels of *Bifidobacterium* spp*.* (Fig. 7B)*, Bacteroides* spp*.* (Fig. 7C)*, Faecalibacterium* spp*.* (Fig. 7D), and *Butyricicoccus* spp (Fig. 7F).

### Perinatal asphyxia results in impaired neonatal behavior, enhanced by prenatal antibiotic administration

#### Experiment 1 (GAA-1)

**Figure 8:**
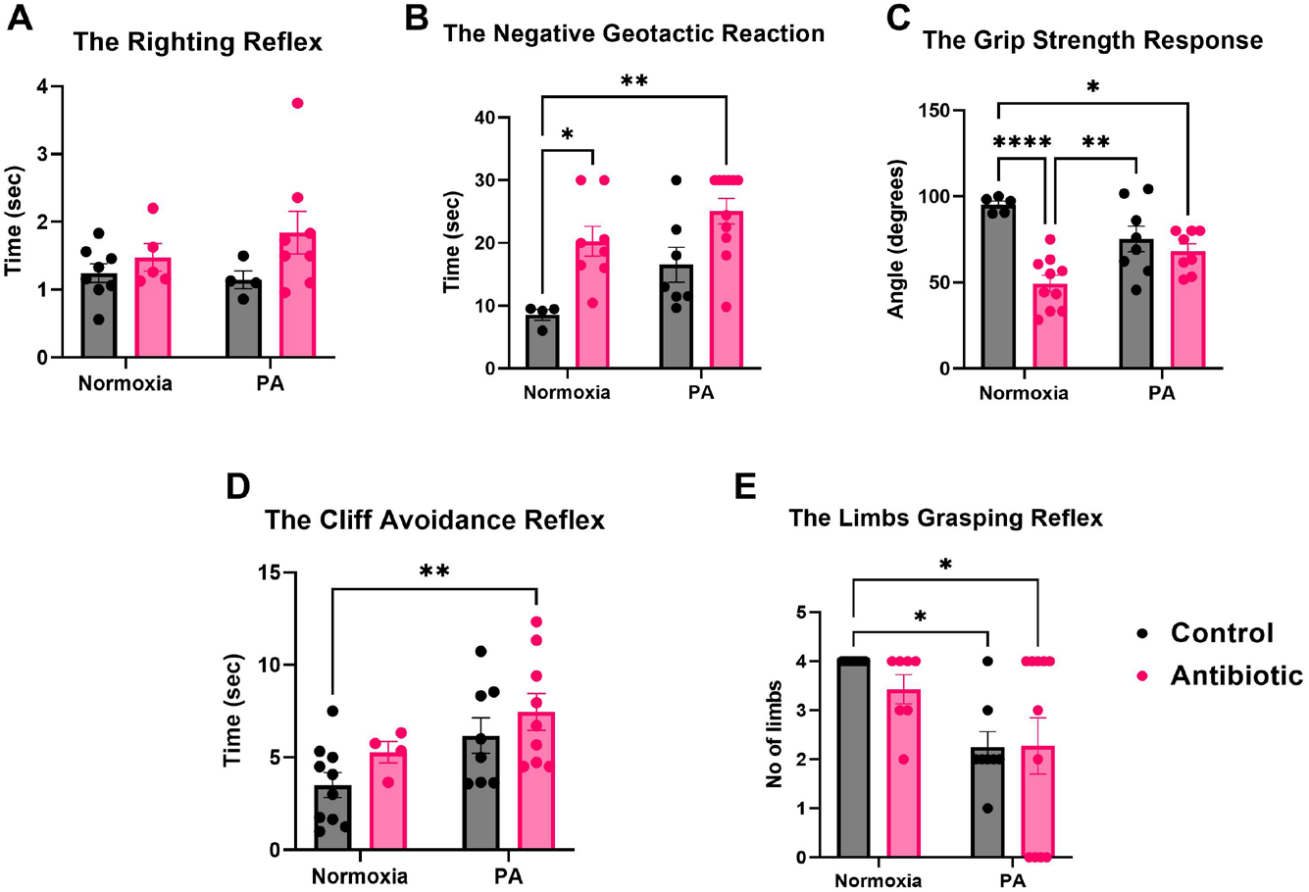
The impact of gestational antibiotic administration (GAA-1) on the offspring neurodevelopmental reflexes in experiment 1. A) In the Righting Reflex test, although not statistically significant, offspring from the AB-N (*n* = 5) and AB-PA (*n* = 8) groups exhibited an extended time to correct themselves compared to the remaining groups (C-N: *n* = 8, C-PA: *n* = 4). B) In the negative geotactic reaction test, significant main effects due to PA (*P* = 0.018) and AB (*P* = 0.0005) were noted; the AB-PA (*n* = 11) group demonstrated the longest latency to turn, followed by the AB-N (*n* = 8) group when compared to the C-N group (*n* = 4). C) In the grip strength response, a significant main effect of AB was found; offspring from the AB-N (*n* = 10) group displayed the weakest paw strength, followed by the AB-PA (*n* = 8) group compared to the C-N (*n* = 5). D) In the cliff avoidance reflex test, a main effect of the PA was noted (*P* = 0.017); pups exposed to both PA and AB (*n* = 9) showed the highest latency in turning away from the cliff compared to control (*n* = 10). E) For the limbs grasping reflex, a main effect of PA was found (*P* = 0.001); both the C-PA (*n* = 8) and the AB-PA (*n* = 11) groups exhibited a notably lower average of successful limb grasps compared to other groups (C-N: *n* = 10, AB-N: *n* = 7). Results are expressed as means ± s.e.m., *P < 0.05, **P = 0.01–0.05, ***P = 0.01–0.001, ****P < 0.001. PA, perinatal asphyxia.

The righting reflex test: although not reaching statistical significance, offspring from the AB-N and AB-PA groups displayed prolonged correction times compared to the control groups, suggesting an overall impact of antibiotic administration (AB-N: 1.476 ± 0.202 sec, *n* = 5, AB-PA: 1.841 ± 0.313 sec, *n* = 8 vs C-N: 1.245 ± 0.138 sec, *n* = 8, C-PA: 1.148 ± 0.132 sec, *n* = 4, *P* > 0.05) (Fig. 8A). The negative geotactic reaction test: PA (*P* = 0.018) and AB (*P* = 0.0005) displayed significant main effects. The AB-PA group exhibited the longest latency to turn, followed by the AB-N group, in comparison to the control group (AB-PA: 25.052 ± 2.030 sec, *n* = 11, AB-N: 20.265 ± 2.396 sec, *n* = 8 vs C-N: 8.538 ± 0.849 sec, *n* = 4, *P* < 0.05) (Fig. 8B).

The grip strength response: a significant main effect of AB was noted (*P* < 0001). Offspring from the AB-N group demonstrated the weakest paw strength, followed by the AB-PA group, in contrast to the C-N group (AB-N: 49.330 ± 4.811°, *n* = 10, AB-PA: 68.123 ± 4.278°, *n* = 8 vs. C-N: 95.264 ± 2.062°, *n* = 5, *P* < 0.05) (Fig. 8C).

The cliff avoidance reflex test: PA exhibited a significant main effect (*P* = 0.017). Pups exposed to both PA and AB displayed the highest latency in turning away from the cliff when compared to the C-N group (AB-PA: 7.460 ± 0.996 sec, *n* = 9 vs. C-N: 3.507 ± 0.674 sec, *n* = 10, *P* < 0.05) (Fig. 8D).

The limbs grasping reflex test: PA again exhibited a significant main effect (*P* = 0.001). Both the C-PA and AB-PA groups demonstrated a significantly lower average of successful limb grasps compared to the control groups (C-PA: 2.250 ± 0.313 limbs, *n* = 8, AB-PA: 2.273 ± 0.574 limbs, *n* = 11 vs C-N: 4 ± 0 limbs, *n* = 10, AB-N:3.429 ± 0.297 limbs, *n* = 7, *P* < 0.05) (Fig. 8E).

#### Experiment 2 (GAA-2)

**Figure 9:**
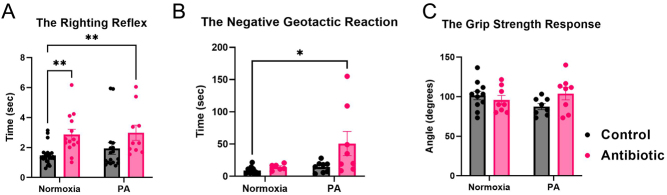
The impact of gestational antibiotic administration (GAA-2) on the offspring neurodevelopmental reflexes in experiment 2. A) In the righting reflex test, AB exposure displayed a significant main effect (*P* = 0.0003); offspring exposed to AB ± PA (AB-N: *n* = 14, AB-PA: *n* = 10) presented with a longer time to flip onto their feet compared to the C-N group (*n* = 26). B) In the negative geotactic reaction, both AB and PA exhibited a significant main effect with *P* = 0.040, and *P* = 0.042, respectively; offspring exposed to both AB and PA (*n* = 8) presented with a longer time to turn up the slope compared to the C-N group (*n* = 10). C) In the grip strength response, no differences between the groups were noted. Results are expressed as means ± SEM, *P < 0.05, **P = 0.01–0.05. PA, perinatal asphyxia.

The righting reflex test: prenatal antibiotics displayed a significant main effect (*P* = 0.0003). Offspring exposed to AB ± PA displayed a significantly prolonged duration to achieve upright posture compared to the C-N group (AB-N: 2.861 ± 0.358 sec, *n* = 14, AB-PA: 2.984 ± 0.503 sec, *n* = 10 vs C-N: 1.476 ± 0.119 sec, *n* = 26, *P* < 0.05) (Fig. 9A).

The negative geotactic reaction test: both AB and PA exposure resulted in significant main effects with *P* = 0.040, and *P* = 0.042, respectively. Offspring exposed to AB and PA demonstrated an extended duration to navigate up the slope compared to the C-N group (AB-PA: 50.819 ± 18.814 sec, *n* = 8 vs C-N: 9.094 ± 1.792 sec, *n* = 10, *P* < 0.05) (Fig. 9B). The grip strength response: There was no significant variability across the experimental groups (*P* > 0.05) (Fig. 9C).

### Antibiotic use during pregnancy (GAA-1) alongside perinatal asphyxia results in increased levels of inflammation and injury in offspring hippocampal tissue homogenate

**Figure 10:**
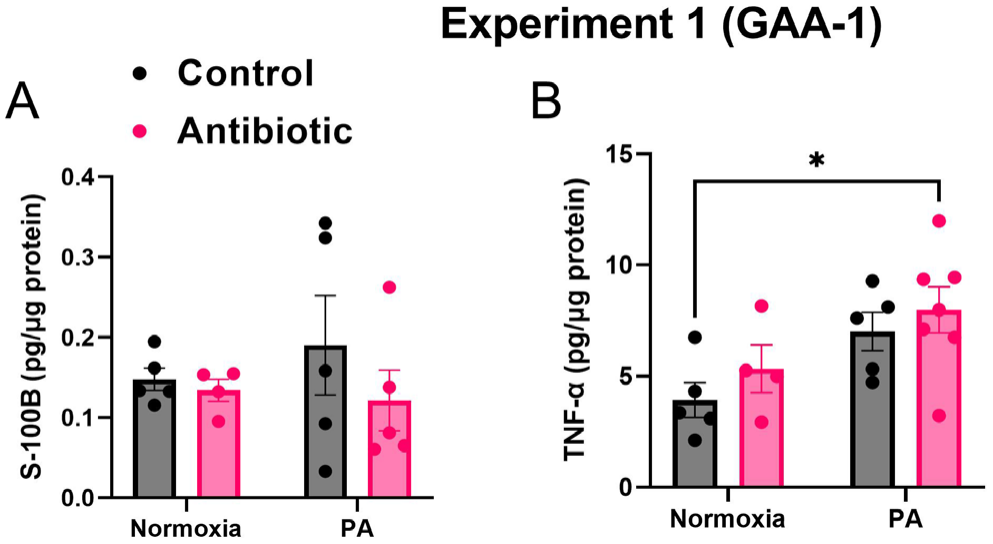
Hippocampal levels of TNF-α and S-100B proteins were assessed 24 h post-asphyxia. Experiment 1: A) GAA-1 didn’t manage to induce significant variability between groups when assessing S- 100B protein levels (*P* > 0.05); B) For the TNF-α levels, PA resulted in a significant main effect (*P* = 0.010); The AB-PA (*n* = 7) group exhibited the highest levels of TNF-α obtained from hippocampal tissue homogenate when compared to the C-N (*n* = 5) group (*P* = 0.031). Results are expressed as means ± SEM, *P < 0.05. GAA, gestational antibiotic administration.

Hippocampal levels of TNF-α and S-100B proteins were assessed 24 h post-asphyxia. In experiment 1, GAA-1 did not induce significant variability between groups in the assessment of S-100B protein levels (*P* > 0.05) (Fig. 10A). However, for TNF-α levels, PA resulted in a significant main effect (*P* = 0.010). Specifically, the AB-PA group exhibited the highest levels of TNF-α obtained from hippocampal tissue homogenate when compared to the C-N group (7.978 ± 1.036 pg/μg, *n* = 7 vs 3.925 ± 0.787 pg/μg, *n* = 5, *P* = 0.031) (Fig. 10B).

In experiment 2 (preliminary data, supplementary data, see section on supplementary materials given at the end of this article), for S-100B protein, AB administration induced a significant main effect (*P* = 0.026). Notably, the AB-N group presented the highest level of S-100B protein levels when compared to the C-N and C-PA groups, although with a limited number of samples (AB-N: 0.269 ± 0.139 pg/μg, *n* = 2 vs C-N: 0.012 ± 0.004 pg/μg, *n* = 3, *P* = 0.048, C-PA: 0.014 ± 0.007 pg/μg, *n* = 4, *P* = 0.040). Regarding TNF-α levels, AB administration resulted in a significant main effect (*P* = 0.028), although no direct variability between each group was observed (*P* > 0.05), considering the limited number of samples.

## Discussion

Maternal antibiotic usage during pregnancy has become increasingly prevalent, serving as a crucial intervention for combating various infections (Broe *et al.* 2014). However, amidst its widespread use, concerns have arisen regarding the potential consequences on pediatric health. This concern is compounded by the escalating global threat of antibiotic resistance, which necessitates a comprehensive understanding of the collateral effects of antibiotic exposure (Galoș *et al.* 2023, Prajescu *et al.* 2023). Epidemiological investigations have unveiled alarming associations between early-life antibiotic exposure and heightened susceptibility to immune and metabolic disorders, underscoring the intricate interplay between antibiotic administration and the delicate developmental trajectories of offspring (Lamont *et al.* 2020). Of particular concern are the neurodevelopmental repercussions linked to maternal antibiotic exposure during pregnancy. Studies have elucidated a troubling correlation between prenatal antibiotic therapy and increased incidences of neurodevelopmental disorders, including attention deficit hyperactivity disorder (ADHD) and epilepsy, underscoring the multifaceted impact of antibiotics on fetal brain development (Tao *et al.* 2022, Straughen *et al.* 2023).

Therefore, considering the known impact of antibiotics on the gut microbiome (Patangia *et al.* 2022), it is plausible to hypothesize that any observed effects on neurodevelopment could be mediated, at least in part, by alterations in the microbial composition of the gut. This assertion is particularly relevant given that antibiotic administration is commonly utilized as a means to induce microbiome depletion, serving as a valuable model for exploring the role of the gut microbiome across diverse physiological contexts (Zarrinpar *et al.* 2014).

In neonatal rodents, maternal antibiotic exposure changes the bacterial communities of the gut microbiota and causes intestinal damage and inflammation by reducing vascularization and cellular proliferation, and impairing the intestinal barrier (Chen *et al.* 2021). Regarding neurodevelopment, the expression of genes related to axonogenesis, synaptic plasticity, and behavior is impaired in rodents from antibiotic-treated dams (Desbonnet *et al.* 2015, Vuong *et al.* 2020, O’Connor *et al.* 2021). Moreover, antibiotic administration to dams results in behavior deficits in the offspring but with conflicting outcomes regarding activity, social interactions, and anxiety (Degroote *et al.* 2016, Tochitani *et al.* 2016, Leclercq *et al.* 2017, O’Connor *et al.* 2021). Furthermore, antibiotic-mediated changes in the gut microbiota can induce a decrease in the level of hypothalamic neuropeptides such as oxytocin (Desbonnet *et al.* 2015), a neurohormone with a fundamental role in maternal behavior (Zagrean *et al.* 2022).

PA, characterized by inadequate blood flow or gas exchange, is linked to elevated mortality and morbidity rates among children (Lawn *et al.* 2011). The therapeutic options for PA and subsequent HIE remain notably restricted, and translating findings from animal studies to clinical settings has proven exceptionally challenging. Despite notable progress in medical advancements, hypothermia remains the primary neuroprotective intervention for term neonates with HIE following PA, as it is the sole therapy supported by substantial clinical evidence demonstrating neuroprotective benefits (Antonucci *et al.* 2014). However, the available data on the effectiveness of hypothermia in reducing mortality and ameliorating neurodevelopmental disabilities among infants affected by PA remains limited (McGuire 2007).

Therefore, it becomes imperative to explore maternal factors that may exert an influence on the outcome of PA in offspring. We have previously investigated several maternal factors that can modulate the severity and impact of PA and found that diet-related factors, such as a high-fat diet (Isac *et al.* 2018) increase vulnerability in the neonate, while other nutrients, such as trans-resveratrol and citicoline, supplemented into the maternal diet provide neuroprotection for the PA exposed offspring (Isac *et al.* 2017, Isac *et al.* 2020). Since diet is the pivotal sculptor of the diversity and composition of the gut microbiota (Ionescu *et al.* 2024), we postulate that the maternal gut microbiome plays a crucial role in determining the outcome of offspring subjected to PA. Thus, it is necessary to develop a model that comprehensively integrates the multifaceted influences of maternal microbial alterations induced by antibiotic administration and subsequent neurodevelopmental outcomes in offspring following PA.

### The adverse effects of the first antibiotic cocktail necessitated refinement of the antibiotic formulation

Our initial experimental findings have provided valuable insights into the specific risks associated with certain antibiotic cocktails, highlighting the need for targeted refinement to mitigate potential harm. The administration of our first antimicrobial cocktail (GAA-1) resulted in poor weight gain during pregnancy, elevated rates of miscarriages, reaching 50%, and reduced offspring weight, prompting a reassessment of the constituents of the cocktail.

It’s noteworthy that we strictly adhered to the ethical standards of the animal facility, ensuring the experiment proceeded as the dams’ weight loss during pregnancy was not more than 20% of the baseline. Additionally, we observed a decline in glucose levels in these animals, likely attributed to reduced intake of water and subsequent food consumption. Although our antimicrobial cocktail composition was based on previous studies that deemed it safe, literature evidence has encountered similar challenges to ours. For instance, a study investigating the effects of antibiotic-induced microbiome depletion on intestinal health utilized a similar antimicrobial cocktail (with metronidazole instead of clindamycin) in drinking water. They observed rodents experiencing weight loss and reduced water intake, leading to the need for a switch to gavage administration (Reikvam *et al.* 2011). Subsequent modifications aimed to optimize pregnancy safety by excluding compounds known for adverse outcomes, such as amphotericin-B and its solvent DMSO, and substituting potentially safer alternatives for antibiotics like clindamycin.

Amphotericin-B can increase the stillbirth rates in rodent and rabbit models, as documented in previous studies (Larson *et al.* 2000). In rabbits, administration of amphotericin-B led to a higher incidence of spontaneous abortions (King *et al.* 1998). Furthermore, research employing a chick embryo model revealed that amphotericin-B impacted angiogenesis, upregulated the expression of apoptosis-related genes, and induced histopathological changes characterized by degenerative and apoptotic-necrotic alterations (Khosravi *et al.* 2022). Given the short-term nature of our antibiotic regimen and lack of fungal overgrowth concerns, we opted to exclude amphotericin-B from our protocol.

Administration of DMSO to pregnant mice resulted in reduced birth weight and decreased serum glucose in males, with females showing decreased hippocampal brain-derived neurotrophic factor expression (Chen *et al.* 2023). DMSO also heightened oxidative stress, inducing apoptosis in mouse embryonic fibroblasts (Wuputra *et al.* 2022). It interfered with early embryonic development in a concentration-dependent manner, potentially arresting development at various stages due to oxidative stress, leading to mitophagy and autophagy. Additionally, DMSO reduced blastocyst quality, impaired implantation capabilities, and increased pregnancy loss (Kang *et al.* 2017) which might explain the high number of stillborn pregnancies recorded during experiment 1. Given these adverse effects and the exclusion of amphotericin-B, DMSO use was deemed unnecessary.

Clindamycin, categorized as FDA pregnancy category B, is generally considered safe for use during pregnancy, although it can cross the placental barrier (Brigg’s Drugs in Pregnancy and Lactation). While animal studies have not shown teratogenic effects (Bollert *et al.* 1974), potential adverse effects on fetal development in humans have been suggested. A Canadian population-based cohort study reported a small increased risk of musculoskeletal malformations and defects in cardiac septum associated with clindamycin use during pregnancy (Muanda *et al.* 2017). Similarly, analysis of the EUROmediCAT database revealed a potential association between clindamycin exposure during pregnancy and hydrocephalus (Leke *et al.* 2021). In a zebrafish model, lincomycin, the parent compound of clindamycin, exhibited neurotoxic effects, including brain deformities and altered neurodevelopmental genes, along with increased acetylcholine esterase activity and oxidative stress (Cheng *et al.* 2020). Given clindamycin’s significant oral bioavailability and its capacity to cross the placental barrier, potentially posing detrimental effects on fetal development, we decided to replace this antibiotic within our formulation.

Notably, the quest for pregnancy-safe antibiotics has led to the identification of oral vancomycin, neomycin, and meropenem as viable options due to their limited systemic absorption and minimized fetal exposure (Fröhlich *et al.* 2016, de Bruijn *et al.* 2020, Raza *et al.* 2021). Additionally, ampicillin has been extensively studied in pregnant women and has not been associated with an increased risk of adverse pregnancy outcomes or birth defects (Bookstaver *et al.* 2015).

Following the utilization of the optimized cocktail, the weight gain during pregnancy was appropriate, with glucose levels comparable to the control group, with no miscarriage occurrences, and with substantial litters with no discernible variations in offspring weights. Therefore, for the subsequent experiments, we opted to employ the second antibiotic cocktail, which was refined based on the observations from the initial trials. The second cocktail was meticulously formulated to strike a balance between achieving microbiome modulation and minimizing potential maternal and offspring health risks.

### Antibiotic administration during pregnancy induced alterations in the maternal gut microbiome

Although the number of samples was limited, efforts were made to assess the influence of the administered antibiotic cocktails on the composition of the maternal gut microbiome in both experiments. In both experiments, the antibiotic-exposed group showed a slight, non-significant reduction in *Butyricicoccus* species compared to the control. *Butyricicoccus* ferments undigested fibers into butyrate, a short-chain fatty acid (SCFA) with anti-inflammatory and immunomodulatory effects (Xi *et al.* 2021, Singh *et al.* 2023). Reduced *Butyricicoccus* levels are linked to neurological and psychiatric disorders, including Postpartum Depressive Disorder (Zhou *et al.* 2020) and ADHD (Wang *et al.* 2023). Butyrate is crucial for neurodevelopment, as low-fiber diets in rodent models lead to reduced butyrate, impaired motor skills, anxiety, and cognitive deficits, while maternal butyrate supplementation improves cognitive function and neuroplasticity (Hernández-Martínez *et al.* 2022). Butyrate's effects on neural proliferation are dose-dependent, with physiological levels being beneficial and high levels causing neurotoxicity (Yoo *et al.* 2011, Yang *et al.* 2020).

Bacteria from the *Faecalibacterium* genus are among the main butyrate-producing microorganisms in the gut (Faden 2022). A lower level of *Faecalibacterium* was found in psychiatric diseases such as major depressive disorder (Jiang *et al.* 2015), ADHD (Wan *et al.* 2020), and autism spectrum disorder (ASD) (Angelis *et al.* 2013). However, studies also identified an augmented proportion of this genus in ASD (Iglesias-Vázquez *et al.* 2020) and epilepsy (Cui *et al.* 2022), highlighting its complex contribution to human pathology. In experiment 2, the antibiotic cocktail decreased the* Faecalibacterium* level, in accordance with a dysbiotic, pro-inflammatory state. Intriguingly, in experiment 1, bacteria from the genus *Faecalibacterium* displayed a significantly higher abundance. However, there is evidence that certain antibiotics can increase the proportion of this genus. For example, antibiotic administration (mainly cephalosporins and erythromycin) during the first year of life was associated with a higher percentage of *Faecalibacterium* (Li *et al.* 2022), and nitrofurantoin had the same effect in patients suffering from urinary tract infections (Stewardson *et al.* 2015). Moreover, the results could be explained by the considerable variability in the behavior of different species, since intestinal inflammation was associated with an increased abundance of *Faecalibacterium longum* L2-6, but a decreased level of *Faecalibacterium duncaniae* A2-165 (Martín *et al.* 2023).

Regarding *Bacteroides* species, our findings yielded conflicting trends between the two experiments, albeit lacking statistical significance. Despite the discordant nature of these findings, they are congruent with existing literature. A rodent study employing ampicillin to induce gestational dysbiosis revealed that male offspring exhibited modified patterns in ultrasonic communication and later reduced social behavior. Examination of the gut microbiota composition indicated an elevation in *Bacteroides*, among other genera (Morel *et al.* 2023). On the other hand, the administration of *Bacteroides fragilis* as a probiotic in a murine model displaying characteristics of ASD improved the behavior and gut permeability (Hsiao *et al.* 2013). Furthermore, a cohort study of 405 infants found a positive correlation between *Bacteroides*-dominant microbiota and better results for cognitive, language, and motor development at the age of two (Tamana *et al.* 2021 Jan 1). Interestingly, another study associated an increase in the abundance of *Bacteroides* with fetal growth restriction (Tu *et al.* 2022). These taken together paint a cloudy picture of the specific importance of *Bacteroides* regarding the offspring’s neurodevelopment.

In both experiments, *Fusobacterium* exhibited elevated levels in the antibiotic-exposed group compared to the control group.* Fusobacterium* recognized as an opportunistic pathogen (Umana *et al.* 2019) was related to adverse pregnancy outcomes and inflammatory bowel disease (Fan *et al.* 2023). Moreover, members of the *Fusobacterium* genus can provoke inflammation by activating pathways such as p-ERK, p-CREB, and NF-κB, particularly in gut microbiota antibiotic depletion scenarios (Engevik *et al.* 2021).

Antibiotic exposure resulted in a marginal reduction of *Lactobacillus* and *Bifidobacterium* levels across both experimental conditions. These bacteria represent some of the most used strains as probiotics. Although they have been shown to reduce cytokine levels, the mechanism remains unclear (Wang *et al.* 2015, Kar *et al.* 2022). The administration of these species to pregnant female rats effectively mitigated the neuroinflammation levels induced by lipopolysaccharides (LPS) (Kar *et al.* 2022). Aside from the inflammatory signaling-reducing properties, the SCFAs produced by *Bifidobacterium* and *Lactobacillus* promote the development of microglia, increase neurogenesis, and strengthen the blood-brain barrier (Spichak *et al.* 2021). The beneficial role of *Bifidobacterium* has been highlighted in multiple studies, being associated with decreased anxiety, stress, depression, and better learning in mice (Savignac *et al.* 2015). That being said, the slightly decreased levels of *Bifidobacterium* and *Lactobacillus* could negatively impact the central nervous system, leading to the impaired neurodevelopmental parameters of the antibiotic cohort.

In experiment 1, exposure to antibiotics resulted in a significant elevation in the levels of *Enterobacteriaceae*, a family of gram-negative bacteria known to produce LPS. The presence of LPS typically triggers the activation of proinflammatory mediators, such as TNF (Jang *et al.* 2018).* Escherichia coli* treatment in mice increases NF-κB activation and TNF-α expression in the hippocampus, which could impact spatial memory and memory consolidation. Additionally, *Escherichia coli* suppresses the expression of tight junction proteins, boosting the absorption of additionally administered LPS, and sustaining its effects (Jang *et al.* 2018). A high abundance of *Escherichia coli* has also been associated with increased hypoxia-ischemia-induced brain injury (Drobyshevsky *et al.* 2024).

A systematic review showed that clindamycin can reduce the abundance of *Lactobacillus, Fusobacterium, Bacteroides,* and *Bifidobacteria*, with similar effects seen for meropenem (Zimmermann & Curtis 2019). Data on ampicillin's impact were limited, but amoxicillin, a related antibiotic, increased levels of *Enterobacteriaceae, Lactobacillus, Bacteroides*, and *Bifidobacteria.* However, amoxicillin increased *Lactobacillus* abundance, while ampicillin reduced it. Another study found that ampicillin or vancomycin increased *Bacteroides* and decreased *Lactobacillus* in mice (Huang *et al.* 2022). Conversely, a combination of these antibiotics with neomycin and metronidazole decreased *Bacteroides*. These findings highlight the complex interactions within the gut microbiota and suggest that antibiotic mixtures can lead to unexpected changes in bacterial abundance.

### PA impaired neurodevelopmental reflexes: a phenomenon exacerbated by maternal antibiotic supplementation

Neurodevelopmental reflex testing serves as a valuable tool in clinical practice to assess nervous system maturation, with aberrant developmental reflexes often indicative of conditions such as cerebral palsy, which may manifest following PA (Nguyen *et al.* 2017). This approach enables the early detection of brain injury and allows for the evaluation of maternal microbiome interventions, which would otherwise be challenging in such young animals.

Each of these neurodevelopmental reflexes assesses different parts of innate behavior. The RR tests the motor ability of the rodent to flip onto its feet from a supine position, testing the vestibular system, and prolonged or loss of righting reflex is associated with impaired neurodevelopment (Panaitescu *et al.* 2018). The NGR is employed to evaluate motor coordination by investigating the automatic vestibular response to geogravitational stimuli. This reflex represents an innate reaction characterized by directional movement against gravitational cues, providing insights into sensory or proprioceptive function. Serving as an initial assessment tool, the NGR aids in the evaluation of motor development, reflexes, activity patterns, and vestibular function (Alberts *et al.* 2004, Ruhela *et al.* 2019). In the GSR, the strength of all four paws is tested. Regarding climbing and sprinting across irregular terrains, a rodent's proficiency in utilizing all four paws for grasping holds significant importance (Feather-Schussler & Ferguson 2016). The CAR is useful for measuring vestibular imbalances and evaluates the labyrinth reflexes, strength, and coordination (Feather-Schussler & Ferguson 2016). A reduced or negative LGR during early life can suggest later development of spasticity, thus indicating damage to the nervous system (Futagi *et al.* 2012).

In both experimental setups, maternal antibiotic administration and PA elicited detrimental effects on early neurodevelopmental parameters. Dysbiosis induced by prenatal interventions was associated with impaired neurodevelopmental reflexes in preclinical studies, and these impairments were reduced by restoring the homeostasis of the gut microbiota (Yuan *et al.* 2022, Chen *et al.* 2024). However, the observed changes in experiment 1 may not solely be attributed to the impact of antibiotics on the intestinal microbiome. Additionally, the potential teratogenic effects of the antimicrobial cocktail components, particularly GAA-1, should be considered.

### PA increased hippocampal inflammation and injury, augmented by antibiotic administration during pregnancy

We previously noted S-100B protein and TNF-α to be indicative biomarkers for evaluating hippocampal damage associated with PA (Isac *et al.* 2017). In experiment 1, neither antibiotic exposure nor PA elicited significant changes, possibly due to the limited sample size or the short 24-h recovery period post-asphyxia. However, TNF-α levels were notably elevated in the PA group, with antibiotics exacerbating this effect. In experiment 2, antibiotic exposure yielded a significant main effect on both S-100B and TNF-α levels, under the reserve of a limited number of samples (supplementary data).

S-100B is a calcium-binding protein localized in a multitude of cells comprising the nervous system, concentrated mainly in the glial cells (Michetti *et al.* 2012). Elevated S-100B levels serve as an indicator for predicting early brain damage in neonates affected by PA (Luo *et al.* 2019) and here we note the levels to be exacerbated by the antibiotic cocktail in experiment 2.

TNF-α stands as a pivotal pro-inflammatory cytokine in pathological contexts, predominantly released by astrocytes and microglia. This *de novo* synthesis of TNF-α represents a significant facet of the neuroinflammatory response linked with various neurological disorders (Olmos and Lladó 2014). We noted elevated TNF-α levels in the PA groups, with the effect further potentiated by maternal antibiotic exposure. These findings underscore the profound influence of maternal antibiotic administration on offspring brain function, exacerbating neuroinflammation following PA.

**Figure 11:**
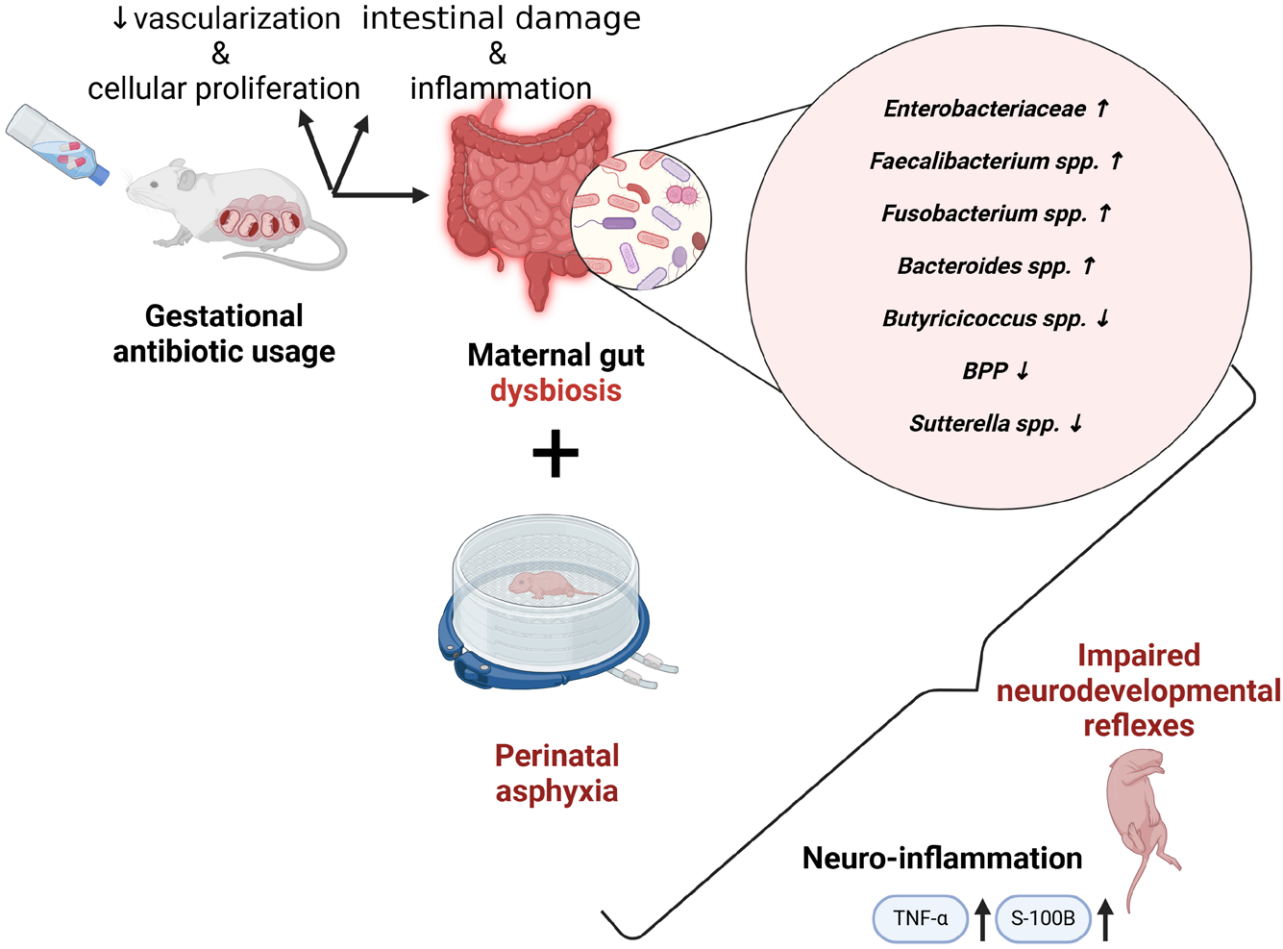
The Link between maternal gut dysbiosis and offspring neurodevelopmental alterations and increased brain vulnerability. This figure illustrates how gestational antibiotic-induced disruption of the maternal gut microbiome leads to dysbiosis. These changes can trigger systemic inflammation and influence fetal brain development. This heightened vulnerability is further exacerbated when the offspring is subjected to PA, resulting in impaired neurodevelopmental outcomes and increased neuroinflammation in the hippocampus.

To summarize, differences have emerged between the groups subjected to gestational antibiotics and those exposed to PA across both experiments. It is imperative to consider that in experiment 1, certain effects attributed to antibiotics may stem from their teratogenic properties rather than solely from microbiome-mediated influences. Consequently, our findings summarized in Fig. 11 suggest that the second antibiotic cocktail represents the most prudent selection for investigating the impact of maternal dysbiosis on offspring subjected to PA.

### Future perspectives

In our ongoing exploration of the microbiota’s impact on the neural development of rat offspring, we plan to enhance our understanding by integrating probiotics alongside the revised antibiotic cocktail. Supplementation with probiotics has shown promise in partially restoring altered microbiota and alleviating some of its effects on the brain (Leclercq *et al.* 2017). Therefore, investigating the potential beneficial effects of probiotic administration following maternal antibiotic treatment aims to elucidate mechanisms underlying probiotic action and their potential to mitigate trans-generational gut-brain axis dysfunction.

In addition to assessing the immediate neurodevelopmental outcomes in offspring, long-term consequences of maternal gut microbiome alterations on both maternal and offspring behavior in the context of PA should be considered. By conducting extended follow-up assessments, the enduring impacts of maternal dysbiosis and antibiotic exposure during pregnancy on various aspects of behavior, including social interactions, cognitive functions, and emotional regulation, particularly in offspring exposed to PA can be revealed. This longitudinal approach can provide valuable insights into the persistence and evolution of neurobehavioral alterations over time, shedding light on the potential trajectories of neurodevelopmental disorders arising from maternal microbiome disruptions exacerbated by PA. Moreover, by examining maternal behavior alongside offspring outcomes, we can uncover the reciprocal influences between maternal gut microbiota composition, PA, and caregiving behaviors, which may further shape offspring neurodevelopmental trajectories.

### Study limitations

It’s important to acknowledge several limitations in our study. First, our sample size may be limited, potentially impacting the generalizability of our findings, especially regarding the microbiome study. Additionally, while rodent models offer valuable insights, they may not fully replicate human physiology and behavior. The duration of our study may also be limited, restricting our ability to assess long-term effects on offspring neurodevelopment. Furthermore, our focus on maternal antibiotic exposure may overlook the potential influence of other maternal factors and environmental variables.

## Conclusion

This study establishes a novel experimental model to investigate the impact of maternal dysbiosis triggered by GAA and combined with PA on offspring neurodevelopment. Maternal gut microbiome disruption induced by GAA-1 resulted in increased miscarriages, poor weight gain during pregnancy, and reduced offspring weight, but refinement of the antibiotic cocktail minimized pregnancy risks. PA further exacerbated neurodevelopmental impairments, augmented by antibiotic exposure. The observed behavioral deficits underscore the significance of maternal factors, particularly gut microbiome integrity, in influencing offspring neurodevelopmental outcomes. These findings emphasize caution in gestational antimicrobial use with subsequent microbiome-associated impairments and warrant further exploration of interventions to mitigate neurodevelopmental sequelae. Future research integrating probiotics alongside antibiotics aims to elucidate protective mechanisms against perinatal brain injury and gut microbiome alterations, contributing to targeted interventions for safeguarding neonatal brain health.

## Supplementary Materials

Supplementary Figure 1

## Declaration of interest

The authors declare that there is no conflict of interest that could be perceived as prejudicing the impartiality of the study reported.

## Funding

This study was funded by Carol Davila Young Investigator Research Grant 1298/31.05.2021 awarded to M.I.I. and by the Sectoral Research and Developmenthttp://dx.doi.org/10.13039/100006190 Program of the Romanian Ministry of National Defensehttp://dx.doi.org/10.13039/501100003559 (PSCD NEURO_Depress 25/2023-2025).

## Author contribution statement

MII, IAD, DB, and C.C. wrote the manuscript. MII, AMC, and GGP performed the data analysis. MII, AMC, CH, VS, and GGP performed the animal behaviour tests and sample processing. SOM and AMZ reviewed the manuscript and supervised the project.
