# Supplementary figures and images for: MICROBIOME: The trials and errors of developing an experimental model to study the impact of maternal gut microbiome disruption on perinatal asphyxia
Source: Reprod Fertil. 2024 Nov 6;5(4):e240050. doi: 10.1530/RAF-24-0050 (PMC11558923; doi:10.1530/RAF-24-0050)

## Experiment 2 (GAA-2)

**A**

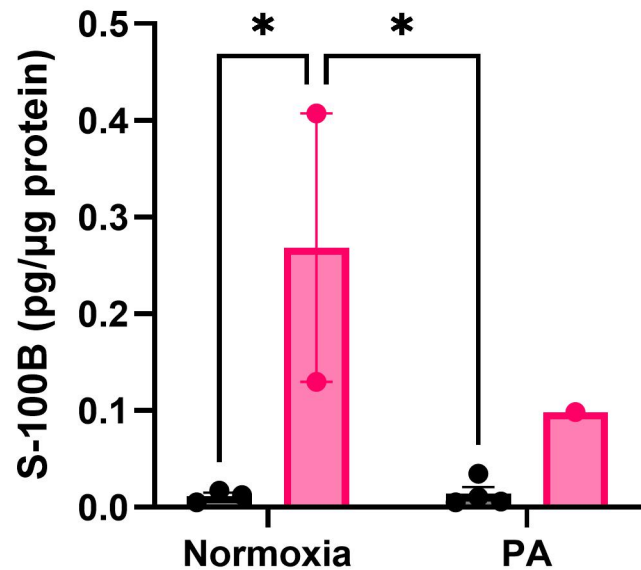

**B**

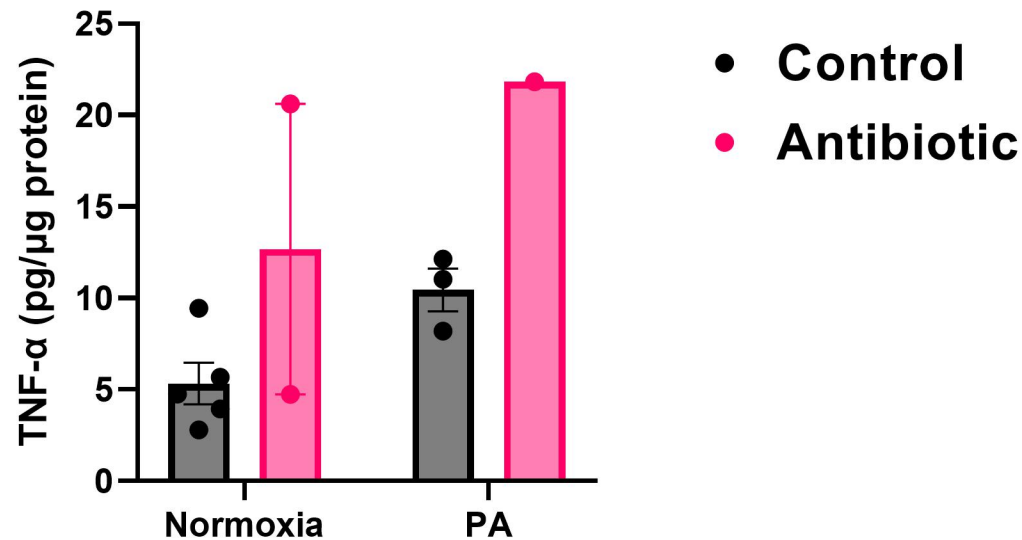

Supplement: Supplementary Figure 1 [file supplementary_figure_1.pdf]
